# The Tribological Properties of Novel Sulfoximine Derivatives as Lubricant Additives

**DOI:** 10.3390/ma17164145

**Published:** 2024-08-22

**Authors:** Jianbin Zhang, Chaoyang Zhang, Yanhua Liu, Libang Feng, Wufang Yang, Xiaowei Pei, Qiangliang Yu

**Affiliations:** 1 School of Materials Science and Engineering, Lanzhou Jiaotong University, Lanzhou 730070, China; jianbin2022@lzjtu.edu.cn (J.Z.); happyliuyanhua@126.com (Y.L.); fenglb@lzjtu.edu.cn (L.F.); 2 Shandong Laboratory of Yantai Advanced Materials and Green Manufacturing, Yantai 264000, China; 18791727994@163.com (C.Z.); ywufang@licp.cas.cn (W.Y.); 3 State Key Laboratory of Solid Lubrication, Lanzhou Institute of Chemical Physics, Chinese Academy of Sciences, Lanzhou 730000, China

**Keywords:** sulfoximine, additives, lubrication, tribofilm

## Abstract

Introducing an additive is a practical approach to improve the lubrication performance of base oil in the field of tribology. Herein, a series of sulfoximine derivatives was synthesized and incorporated into base oil A51 as additives. The tribological properties of these lubricants were evaluated at both room and high temperatures, and the result demonstrated that they displayed excellent friction reduction and wear resistance in the friction process under both test conditions. Moreover, the chemical composition of the worn scar surface was inspected using EDS, XPS and TOF-SIMS to explore the lubricating mechanism. It is reasonable to conclude that the synergistic interaction between the aromatic ring scaffolds and elements like N, F, and S facilitated the adsorption of lubricant on the steel block surfaces and forming a tribofilm during the friction process. This tribofilm has a dominant impact on the system’s lubrication performance. This research provides novel oil-soluble lubricant additives, offering a facile approach to formulating high-quality lubricants.

## 1. Introduction

Friction is an issue that is closely related to different human developments, including clothing, food, housing, and transportation and widely exists in industries such as transportation, manufacturing, energy, and municipal engineering. Therefore, friction and wear are the most common and extensive failure modes of mechanical equipment, and statistical data show that friction consumes 30% of the world’s disposable energy, of which approximately 80% is used for the remanufacturing of part and equipment wear caused by friction [1,2,3]. Researchers have proposed a variety of lubricating materials, including lubricating oils [4,5], lubricating esters [6,7,8], ionic liquid lubricants [9,10,11], lubricant additives [12,13], and lubricating coatings [14,15,16], over the last decade.

Among these lubricating materials, lubricating oils are vital in tribological research, ranging from animal and plant fats to mineral oils [17]. It is reasonable to assume that the use of lubricating oil has accompanied the development of tribology. Early lubricating oils mainly contained saturated fatty acids and long-chain alkanes [18]. However, two main factors hinder the widespread application of these pure base oils. Firstly, the molecular chain of base oils will inevitably undergo cracking under the effect of temperature, pressure and mechanical interactions, resulting in lubrication failure [19]. Secondly, they also inevitably experience a certain degree of crawling and inevitable losses on the moving surfaces of equipment.

Moreover, some lubricating base oils can corrode metal pairings during friction [20,21]. These issues have promoted the emergence of various lubricating additives. Additives are critical in tailoring lubricant properties to meet specific performance requirements under diverse operating conditions. The additives are meticulously designed, and a tiny number of them have been integrated into lubricant formulations to achieve desirable functions, such as improved wear protection, oxidation resistance, thermal stability, viscosity control, and corrosion inhibition [21]. Their unique chemical and physical interactions with base oils and friction pair additives mitigate the adverse effects of friction and wear, thereby ensuring ideal operation, prolonging the service life of machinery components and reducing maintenance costs [19,22]. Therefore, additives are essential for significantly optimizing the performance of base oils between moving surfaces in machinery and adapting to varying operating conditions. Researchers have recently proposed various additives, such as metal nanoparticles, nanometal oxides, and nanocarbon materials [18,23,24]. These studies have greatly encouraged lubrication theory and provided many unique insights into lubricating additives. These exciting works demonstrated that these fascinating additives dispersed in the base oil can significantly enhance the anti-wear and anti-friction properties of the lubricating system [23,25]. However, solid nanomaterials are prone to agglomeration due to their large specific surface area and often require special pretreatment to be evenly dispersed in the base oil. These problems will lead to increased energy consumption in the preparation process and deteriorate the comprehensive performance of lubricating oil.

Compared with solid nano additives, some unique organic compounds, organometallic compounds, and polymers have been adopted as additives for use in base oil, which was an ideal approach to solving the difficulty of dispersion [26,27]. Through molecular design and common synthesis methods, good compatibility with base oil and perfect comprehensive performance were feasible. Much enlightening research has been conducted in recent years. Zinc dialkyl dithiophosphate (ZDDP) is widely used in mechanical equipment due to its antioxidant, anti-wear, and excellent pressure properties. However, later research found that ZDDP can make automobile exhaust treatment catalysts ineffective, which is not conducive to environmental protection [28,29]. Due to N, S, and P elements readily reacting with metals during friction, many lubricant additives with single-element and multi-element synergistic effects have been developed [9,30]. Among them, ionic liquids have attracted widespread attention due to their unique charge properties, low volatility, and designable components [31]. Wang and co-workers synthesized a phosphate ionic liquid containing a long hydrocarbon chain in both anion and cation components and achieved excellent solubility in various base oil and improved the anti-wear ability in poly-alpha-olefin (PAO) [32]. Cai and her co-workers designed two ionic liquids with the same anion (sulfonate) and different cations (quaternary ammonium or quaternary phosphonium cation) named N88816-Doss (N/S) and P88816-Doss (P/S); they systematically studied the tribological properties of these two types of ionic liquids compared with traditional lubricating additives containing sulfur or phosphorus (T321, T204, and T306). The experiment result showed that N/S significantly improved the physicochemical and frictional properties of PAO10 [33]. These substances containing sulfur, nitrogen, and phosphorus are suitable for lubricant applications due to their mechanisms related to the formation of a transition layer and surface passivation [31,32,33]. However, their large-scale use is harmful to the environment. Therefore, some researchers have applied vegetable oils, their polymers, and biomass substances as alternative additives [34,35,36]. There was a representative work that proposed water-based lubricant additives (phosphorus-containing ricinoleic acid and sulfur-containing ricinoleic acid), and the result showed that the synergistic effect of long aliphatic chains and highly active phosphorus and sulfur elements was prone to lubrication [37]. The increasing focus on diminishing the negative impact on the environment has driven efforts to develop environmentally benign lubricating oil additives, and it is necessary to develop novel additives with less nitrogen and sulfur or even no nitrogen and sulfur in the future [38].

Inspired by the above research, sulfoximine, widely used in chemical engineering, catalysis, and pharmaceuticals, was selected as the structural backbone. Compounds with S=N double bonds and large π-bonds were meticulously designed and synthesized as additives. These additives were then employed to formulate three distinct oil-soluble lubricant additives using A51 base oil. The anti-wear and friction-reducing properties of the formulated additives were rigorously evaluated at both room temperature and high temperature (100 °C). Furthermore, the lubrication mechanisms were elucidated through comprehensive analyses of the chemical composition of the wear scar surfaces. This study delineates a novel approach for synthesizing oil-soluble ionic additives with small amounts of S and N elements to improve the lubrication conditions for metal surfaces.

## 2. Materials and Methods

### 2.1. Materials and Chemical Reagents

Trifluoromethanesulfonamide (99%), trifluoroacetic anhydride (TFAA, chemical purity), and benzoyl chloride were provided by Meryer Chemical Technology Co., Ltd. (Shanghai, China). The dibenzo [b, d] thiophene 5-oxide (analytically pure) and dibenzo [b, d] thiophene (analytically pure) were supplied by J&K Chemical Ltd. (Shanghai, China). The ethyl (Z)-N-((mesitylsulfonyl) oxy) acetimidate and trifluoroacetic acid were sourced from the Energy Chemical Reagent Co., Ltd. (Shanghai, China). The solvent, N, N-Dimethylformamide (DMF), dichloromethane (DCM) and acetonitrile (CH3CN, analytically pure), were purchased from Lian-Long Bohua (Tianjin, China) Pharmaceutical Chemical Co., Ltd. (Tianjin, China). Sulfonimidoyldibenzene (SIMDB) was commercially available and was purchased from the Shanghai Jizhi Biochemical Technology Co., Ltd. (Shanghai, China). It was named compound A for the sake of convenient description in the later text. Unless specified, all of the reagents were used in their received condition without further purification. EsterexA51, produced by Mobil Corp. (Springwoods Village Parkway, Spring, TX, USA), was adopted as the base oil. The steel balls and plates used in the friction test were made of AISI 52100.

### 2.2. Preparation of the Lubricant Additives

**Scheme 1:**
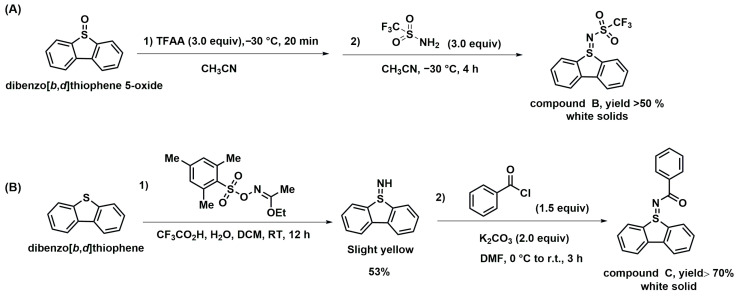
The schematic diagram of the synthetic route of two sulfoximine derivatives as lubricating additives for compound B (**A**) and compound C (**B**).

This research chose three kinds of sulfoximine derivatives as lubricant additives to investigate their lubrication performances on metal surfaces. One (SIMDB) of them is commercially available, and the other two were synthesized according to the reference [39,40,41]. Specifically, as illustrated in Scheme 1A, the synthesis of compound B involved several sequential steps. Initially, dibenzo [b, d] thiophene 5-oxide and TFAA (molar ratio of 1:3) were dispersed with CH_3_CN within a round-bottom flask, and the resulting mixture was stirred at −30 °C for 20 min to disperse uniformly using a magnetic stirrer (DHJF-8002). Subsequently, the powder of trifluoromethanesulfonamide, equivalent to three times dibenzo [b, d] thiophene 5-oxide, was dispersed in a specific solvent and gradually added dropwise to the flask. When the liquid was added and finished, the reaction proceeded for 4 h. Finally, the reaction mixture underwent vacuum distillation at 40℃, with dichloromethane (DCM) serving as the solvent for recrystallization, producing a white powder identified as compound B.

The other was synthesized according to Scheme 1B. The dibenzo [b, d] thiophene was first dissolved with DCM in a round-bottom flask. Afterwards, the powder of ethyl (Z)-N-((mesitylsulfonyl) oxy) acetimidate (in a 1:1 molar ratio), dissolved with the right amount of DCM, was transferred to the flask. Simultaneously, a suitable trifluoroacetic acid was added as a catalyst, allowing the reaction to proceed at room temperature for 12 h. When the reaction was finished, a light-yellow powder (5H-5λ4-dibenzo [b, d] thiophen-5-imine) was prepared via vacuum distillation at room temperature. Subsequently, a certain amount of light-yellow powder was dissolved in DMF, and a 1.5-time equivalent liquid of benzoyl chloride was introduced into the solution. The reaction was performed for 3 h in the presence of K_2_CO_3_, and the temperature ranged from 0 °C to room temperature during this process. Finally, we added a fixed volume of distilled water to the system, yielding a white substance. After post-processing, including separation and recrystallization, a high-purity white solid was obtained, which was named compound C.

## 3. Results

### 3.1. Synthesis of Lubricant Additives

**Figure 1:**
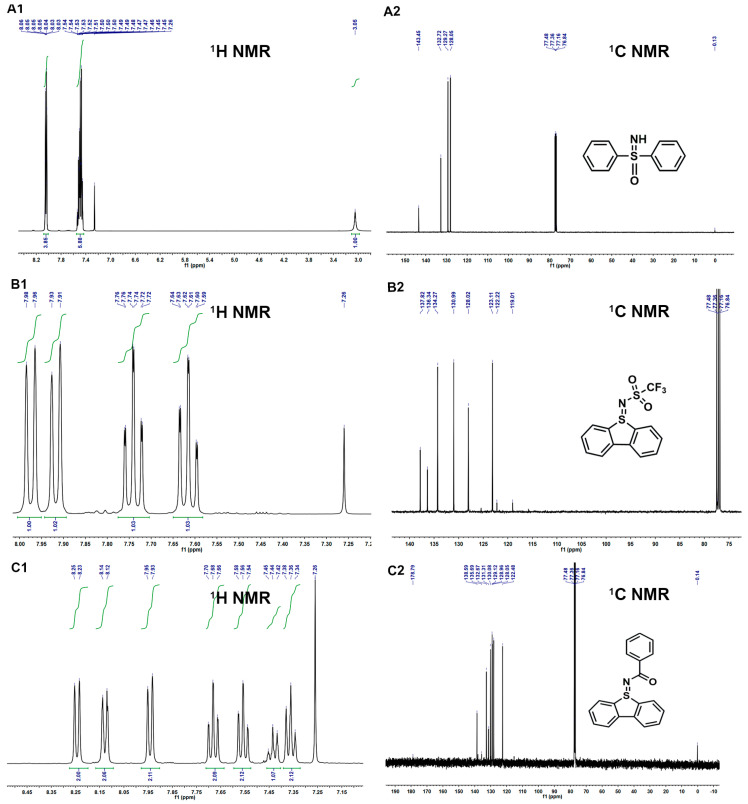
NMR spectra of the lubricant additives for A (**A**), B (**B**), and C (**C**). Character 1 shows the hydrogen spectrum, character 2 represents the carbon spectrum, and the insets in the figure indicate the molecular formula of the additives.

To further confirm the correct synthesis of the three lubricant additives. The molecular structures were characterized using nuclear magnetic resonance (NMR) and mass spectrometry (MS). The NMR spectra are provided in Figure 1, and the chemical shifts were as follows:

A: ^1^H NMR (400 MHz, CDCl_3_), δ: 8.06–8.03 (m, 4H), 7.54–7.45 (m, 6H), 3.05 (s, 1H). ^13^C NMR (100 MHz, CDCl_3_), 143.45, 132.72, 129.27, 128.05. m/z (ESI, positive ion) calc. 218.0634, found: 218.0631 [C_12_H_12_NOS]^+^.

B: ^1^H NMR (400 MHz, CDCl_3_), δ: 7.97 (d, *J* = 4.0 Hz, 2H), 7.92 (d, *J* = 4.0 Hz, 2H), 7.74 (t, *J* = 8.0 Hz, 2H), 7.61 (t, *J* = 8.0 Hz, 2H). ^13^C NMR (100 MHz, CDCl_3_) δ: 137.82, 136.34, 134.27, 130.99, 128.02, 123.11, 122.22, 119.01. m/z (ESI, positive ion) calc. 332.0021, found: 332.0009, [C_13_H_9_F_3_NO_2_S_2_]^+^.

C: ^1^H NMR (400 MHz, CDCl_3_), δ: 8.24 (d, *J* = 8.0 Hz, 2H), 8.13 (d, *J* = 8.0 Hz, 2H), 7.94 (d, *J* = 8.0 Hz, 2H), 7.68 (t, *J* = 8.0 Hz, 2H), 7.56 (t, *J* = 8.0 Hz, 2H), 7.42 (t, *J* = 8.0 Hz, 1H), 7.36 (t, *J* = 8.0 Hz, 2H). ^13^C NMR (100 MHz, CDCl_3_) δ: 178.79, 138.59, 135.69, 132.67, 131.31, 130.08, 129.12, 128.96, 128.05, 122.48. m/z (ESI, positive ion) calc. 304.0791, found: 304.0795 [C_19_H_14_NOS]^+^.

### 3.2. Tribological Studies and Characterization

When the synthesis of the lubricating additives was successfully finished, they were added to the A51 base oil with a 1% mass fraction to prepare the lubricant, which was represented by the additives for the convenience of subsequent description. The tribological properties were examined under room temperature (25 °C) and high temperature (100 °C) for 30 min using the SRV-IV fretting tribological wear tester from Optimol Instruments Prüftechnik GmbH (München, Germany). When tested, a 100 N load was applied, the frequency was 25 Hz, and the amplitude was 1 mm. The upper test ball consisted of AISI 52100 steel with a diameter of 10 mm and a hardness of about 59~61 HRC, while a AISI 52100 steel disc with a diameter of 24 mm was used as the lower test disc; its thickness was 7.9 mm, and it had a hardness of about of 59~61 HRC. Each sample was tested three times to avoid accidental errors, and the average of the three friction coefficients was considered the final result. When the test was finished, to further evaluate the anti-wear and reduced-friction properties, the wear spot morphology and wear volume of the lower steel disc were measured using a BRUKERNPFLEX 3D optical surface profiler (Billerica, MA, USA). Three random points were selected for each sample to determine the wear volume, and their average value was recorded.

### 3.3. Lubrication Mechanism Analysis

In order to investigate the lubrication mechanisms further, various surface analysis techniques were applied in combination to analyze the chemical compositions of the wear spot surfaces. The test plate was ultrasonically cleaned with petroleum ether and acetone, respectively, to remove the lubricating oil physically adsorbed on the surface prior to the characterization of the surface. The morphology and elemental analysis of the wear spot surface were characterized using FEI Quanta 250 SEM/EDS (Hillsboro, OR, USA). In addition, the chemical compositions of the elements on the wear spot surface were analyzed using the Thermo Scientific Nexsa multifunctional X-ray photoelectron spectrometer (XPS) (Waltham, MA, USA), and Al-Kα was selected as the excitation source. Meanwhile, to more accurately analyze the chemical reaction of the lubricant during the friction process, the chemical composition of the wear spot surface was detected through the use of time-of-flight secondary ion mass spectrometry (TOF-SIMS) (Billerica, MA, USA) in positive and negative ion modes.

## 4. Discussion

### 4.1. Tribological Studies

**Figure 2:**
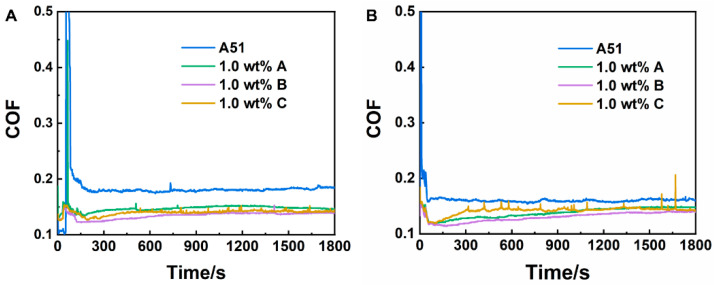
The friction curve and coefficient of friction (COF) of the lubricant with lubricant additives at high and room temperature (**A**) and high temperature (**B**). The applied load was 100 N, the frequency was 25 Hz, and the amplitude was 1 mm during the tribological test.

As shown in Figure 2, all three lubricant additives exhibited excellent anti-friction performance at both room and high temperatures. The friction curves were generally smooth, with minimal jump points during the test. Compared to the A51 base oil, the lubricant additive at a 1% concentration significantly reduces the coefficient of friction (COF). At room temperature, the COF of the base oil was 0.18, while the COFs of the lubricants containing subsequent additives were 0.15, 0.13, and 0.14 for A, B and C (Figure 2A). Under high-temperature conditions, the base oil had a COF of 0.16, while the COFs for additives A and C were about 0.14; additive B had the lowest COF of 0.13 (Figure 2B). The slightly lower COF at high temperatures may be attributed to reduced lubricating oil viscosity and increased susceptibility to frictional chemical reactions. These results demonstrated that trace lubricant additives significantly enhanced the lubricating performance of the friction counterpart.

**Figure 3:**
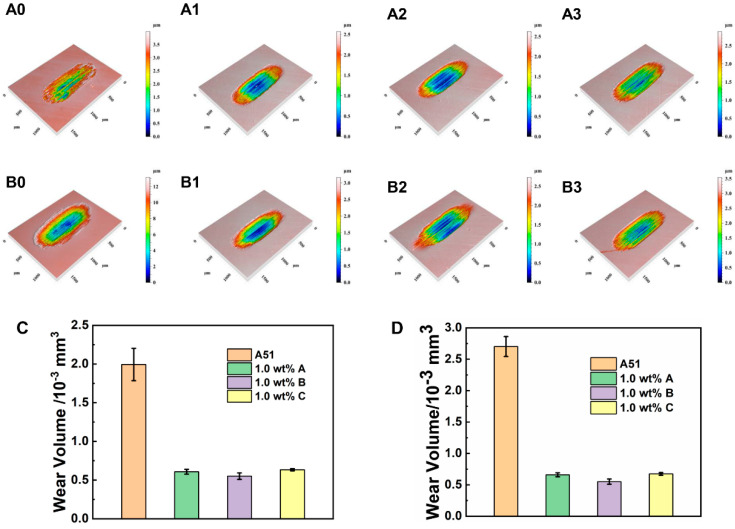
Three-dimensional topographic images (**A**,**B**) of lubricated worn plates and their wear volume statistics (**C**,**D**): (0) A51, (1) 1% A, (2) 1% B, and (3) 1% C. Characters (**A**,**C**) indicate room temperature; characters (**B**,**D**) indicate high temperature.

To further evaluate the anti-friction performance of lubricating additives, the volume of wear spots on the surface of the steel disc was characterized. The results are depicted in Figure 3. It is evident that the additives significantly mitigated wear volume regardless of the test temperature, as shown in Figure 3A,B. The statistical data on wear volume are shown in Figure 3C,D. The wear volume of the A51 base oil was 2 × 10^−3^ mm^3^, and the counterparts of lubricant containing additives were reduced to 0.61, 0.55, and 0.63 × 10^−3^ mm^3^, respectively. This reduction in wear volume reached between one-third and one-quarter of A51 due to the presence of additives. For high temperatures, the wear volume of the base oil increased to 2.7 × 10^−3^ mm^3^, whereas the lubricating oil containing additives exhibited no substantial increase. Notably, for additive B, which demonstrated the most effective anti-friction performance, the wear volume remained at 0.55 × 10^−3^ mm^3^. These findings indicated that lubricating oils containing additives possessed superior anti-friction properties at room and high temperatures, which implied their significant potential for use in mechanical equipment under operating conditions that experience large temperature changes.

### 4.2. Lubrication Mechanism Analysis

**Figure 4:**
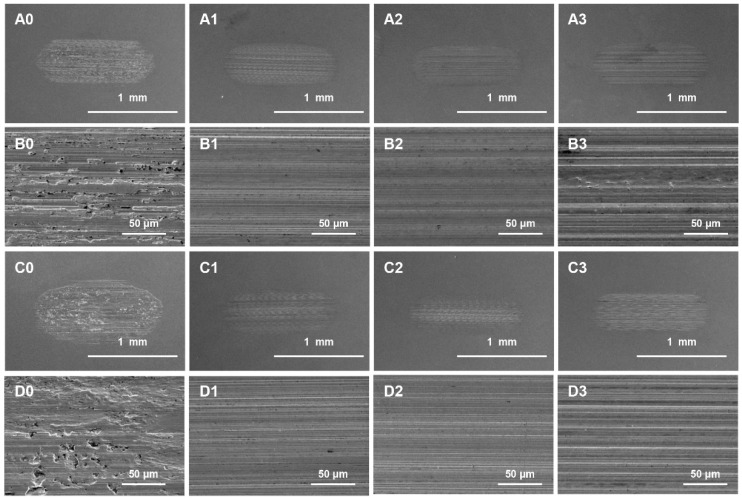
The SEM images of worn scars on steel plates lubricated by (0) A51, (1) 1% A, (2) 1% B, and (3) 1% C. (**A**,**B**) (greater magnification) represent room temperature, and (**C**,**D**) represent high temperature.

The microtopography and surface chemical composition of the wear scar were meticulously examined to investigate the lubrication mechanisms of additives further. As shown in Figure 4, the microstructure of the worn scar surface was similar for the same lubricant at both room and high temperatures. The surface lubricated using the A51 base oil presented a large wear spot diameter, with deep and wide wear marks containing numerous severe grooving and irregular defects indicative of severe adhesive and abrasive wear. The steel plate suffered severe scuffing damage (Figure 4(A0,B0)). In contrast, samples with additives presented smaller wear spot diameters and shallower and narrower wear marks, demonstrating superior lubrication and effective anti-wear properties (Figure 4(A1–A3,B1–B3,C1–C3,D1–D3)). When the additives were added to A51, scuffing damage was significantly reduced, and the worn surface was relatively smooth and dominated by mild abrasive wear (Figure 4(B1–B3,D1–D3)). For example, when using additive B, the worn scar was the smallest and smoothest (Figure 4(A2–D2)), aligning with its lowest friction coefficient and wear volume discussed in Figure 2 and Figure 3.

**Figure 5:**
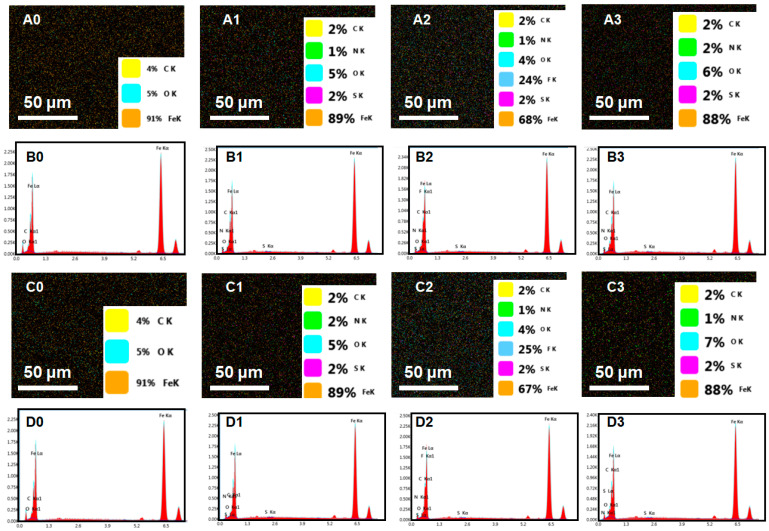
Chemical elemental analysis of worn scar lubricated by (0) A51, (1) 1% A additive, (2) 1% B additive, and (3) 1% C additive. Surface element distribution diagram (**A**,**C**) of area scan and line scan scanning element (**B**,**D**) at the center line of the wear spot. (**A**,**B**) indicate room temperature, and (**C**,**D**) indicate high temperature.

After analyzing the surface morphology of the wear scar, EDS was employed to map the elemental distribution on its surface (Figure 5). Only the intrinsic elements C, O, and Fe of steel were detected for the base oil (Figure 5(A0–D0)). For additives A and C, their characteristic elements, N and S, appeared in the element distribution map (Figure 5(A1–D1, A3–D3)), while for additive B, elements N, S, and F appeared (Figure 5(A2–D2)). These elements were readily adsorbed to some metal surfaces and underwent chemical reactions during friction, leading to the development of lubricating films and preventing direct contact between the friction pairs. At high temperatures, the content of these characteristic elements on the surface of the worn scar was slightly higher than at room temperature, indicating that an appropriate temperature facilitated the reaction of frictional chemical reactions (Figure 5(A1,C1,A2,C2,A3,C3)). This correlation aligned with the pattern shown in Figure 2, where the COF of the same lubricating additives is lower at higher temperatures than at lower temperatures.

**Figure 6:**
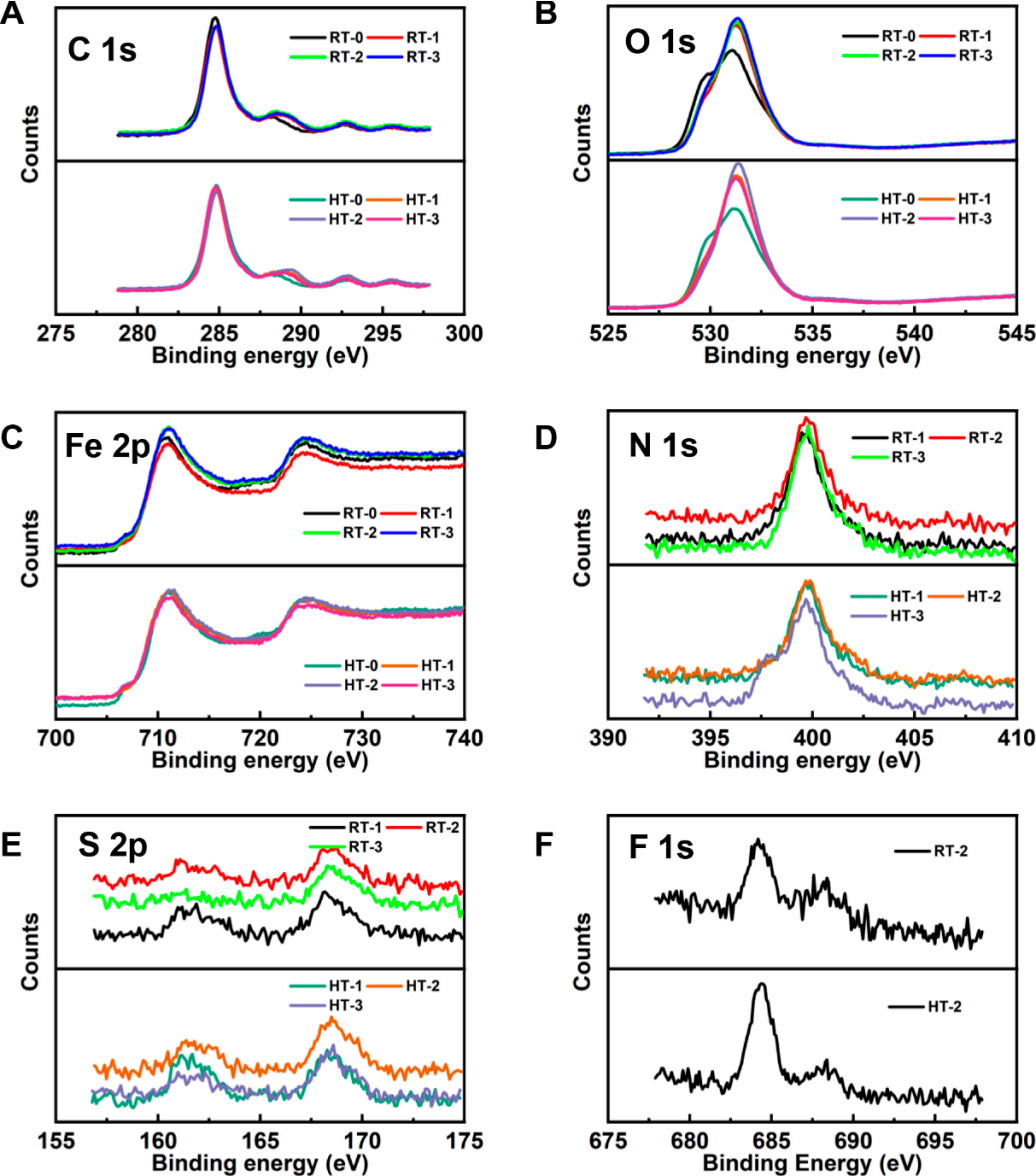
XPS profiles of the worn surface for C 1s (**A**), N 1s (**B**), Fe 2p (**C**), N 1s (**D**), S 2p (**E**), and F 1s (**F**). The characters 0, 1, 2, and 3 represented A51 base oil, additives A, B, and C; RT indicates room temperature; HT indicates high temperature.

The chemical valence states of the characteristic elements on the worn scar surface were analyzed using XPS. The XPS spectra of C 1s, Fe 2p, O 1s, N 1s, S 2p, and F 1s of the worn scars are illustrated in Figure 6. The data shown in Figure 6 revealed that the peak shapes and binding energies of the corresponding elements on the worn scar surface were identical at both room and high temperatures, and this relationship confirmed that the lubricant with the synthetic additive underwent similar tribochemical reaction processes within these temperature ranges.

The C 1s peak can evolve into three peaks located at 284.8 eV, 288.38 eV, and 292 eV, and they correspond to the C-O band, C=O band, and other organic compounds, respectively (Figure 6A). The peak of N1s was around 399.78 eV and was attributed to nitrogen-containing organic compounds or nitrogen oxides (Figure 6D). The prominent absorption peak of O 1s at a binding energy of 530 eV correlated with CO, NO, and metal oxides (Figure 6B). These oxides and organic compounds may result from the adsorption of the alkyl chain fragments of the lubricating oil during friction [32,33]. By combining the Fe 2p peaks at 711.08 eV and 724.58 eV with the S 2p peaks at 168.58 eV and 161.38 eV, it can be inferred that sulfur compounds, such as FeS and FeS_2_, were generated during friction (Figure 6C,E) [42]. Additionally, by correlating the absorption features of Fe 2p with the O 1s absorption peaks, it was reasonable to conclude that a variety of compounds, including Fe(CO)_5_, FeO, Fe_2_O_3_, and Fe_3_O_4_, were present on the surface of the worn scar [33]. Furthermore, for additive B, which contains fluorine elements, the F 1s absorption peaks were decomposed into 684.28 eV and 688.38 Ev (Figure 6F). When these are associated with the Fe 2p absorption spectra, it can be inferred that FeF_2_ and FeF_3_ existed in the tribofilm [43]. These discussions indicated that the sulfides and iron oxides generated on the surface of the steel disc during friction endowed the lubricating additive with excellent lubrication performance. They played a vital role in reducing friction and anti-wear.

**Figure 7:**
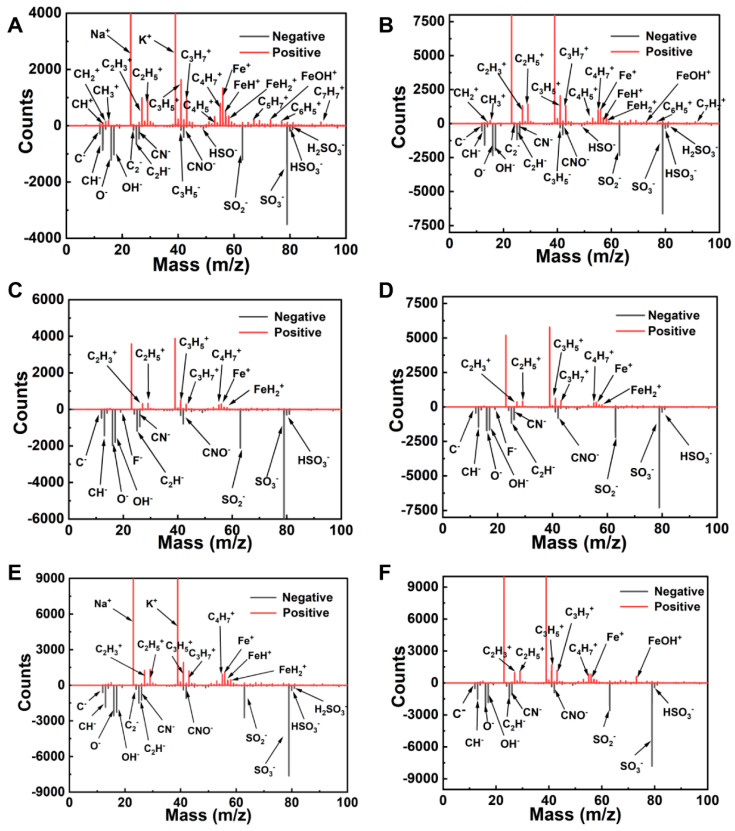
TOF-SIMS mass spectrometry of positive and negative ions generated by tribofilm on worn steel surfaces lubricated with lubricants containing compound A (**A**,**B**), compound B (**C**,**D**), and compound C (**E**,**F**). The icons (**A**,**C**,**E**) indicate room temperature; (**B**,**D**,**F**) indicate high temperature.

TOF-SIMS is often used to characterize the chemical composition of functional films due to its high sensitivity and accuracy. In this study, the valence states of the characteristic elements were analyzed through the use of XPS and integrated with TOF-SIMS to inspect the material composition of the tribofilm formed by the lubricating additives during the friction process. As shown in Figure 7, different mass-to-charge ratios represented different fragment ions in mass spectrometry, and some critical fragment ions have been assigned to the figure. For the same additive, the fragment ions on the surface of the lubricating film generated during high- and low-temperature friction tests were basically the same.

The positive spectrum displayed many hydrocarbon clusters (C_x_H_y_), indicating that the long-chain alkyl chains of the lubricating oil cracked during the friction process and participated in the formation of the tribofilm. Cations fragments, such as Fe^+^, FeH^+^, FeH^2+^, and FeOH^+^, were also detected, and high-intensity ions with aromatic structures also appeared at m/z 77, 91. The anions were mainly fragments of carbides, nitrides, and sulfides, including C_2_^−^, O^−^, OH^−^, CN^−^, CNO^−^, SO_2_^−^, and SO_3_^−^ of the negative spectrum (Figure 7A,B,E,F). Especially for lubricant additive B, the mass ion of F- was present (Figure 7C,D). These findings implied that lubricating oils containing additives formed a critical lubricating film during friction, and this film was primarily composed of nitrides, sulfides, and iron oxides. These insights aligned closely with the results of the XPS analysis.

**Figure 8:**
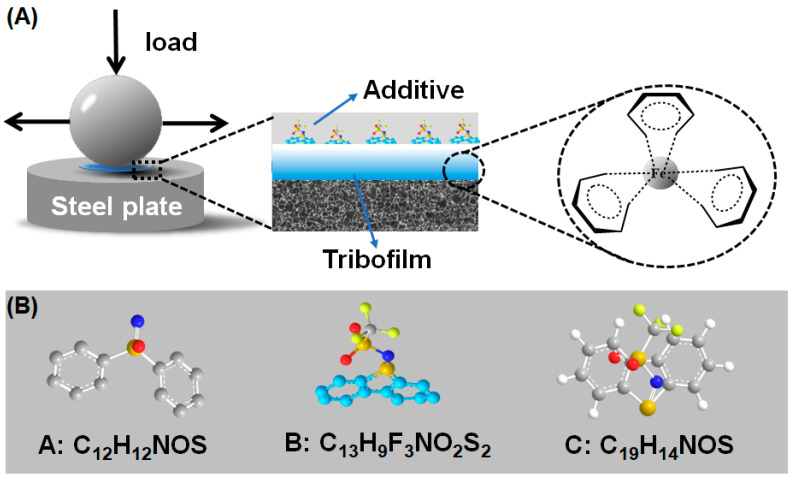
Schematic diagram of the lubrication mechanism of a lubricant containing synthetic additives. The formation diagram of tribofilm during friction process (**A**) and the molecular structure of the additives (**B**).

Based on the above analysis, it is reasonable to conclude that the outstanding tribological performances of the lubricants containing additives were attributed to the formation of a complex of tribochemical reaction products, such as sulfide, oxide, and nitrogen compounds, during friction. The lubrication mechanism is illustrated in Figure 8, and the abundant delocalized π electrons in the aromatic ring structure of the additive were prone to coordination with metal empty orbitals, even orientation [12,13]. Furthermore, elements such as N, S, and F were also easily absorbed to the surface of the metal; afterward, they developed tribofilms containing sulfide and Fe oxide complexes, preventing direct contact between the friction pairs and endowing the entire system with good lubrication [44].

## 5. Conclusions

This research synthesized a series of sulfoximine derivatives and added them to A51 base oil as additives to prepare lubricants. Their tribological behaviors were evaluated at both room and high temperatures, and the results indicated that these aromatic ring compounds containing N, S, and F elements could notably enhance the friction-reducing and anti-wear properties of base oils. Subsequently, some surface analysis techniques, such as EDS, XPS, and TOF-SIMS, were employed to characterize the chemical compositions of the worn surfaces and investigate the related lubrication mechanisms. It is reasonable to conclude that these additives were readily adsorbed onto the surfaces of steel plates and underwent tribochemical reactions during friction, resulting in the formation of a tribofilm composed of sulfides and iron oxides. This tribofilm played a crucial role in improving the lubrication performance of the base oil. This research paves the way for novel applications of sulfoximine compounds and proposes a novel method for preparing a high-performance lubricating oil with excellent application potential. This research also provided a valuable insight for developing lubricating oil additives.

## Data Availability

All data generated or analyzed during this study are included in this manuscript.
